# Supplementary material for: Plantago asiatica Seed Extracts Alleviated Blood Pressure in Phase I–Spontaneous Hypertension Rats
Source: Molecules. 2019 May 4;24(9):1734. doi: 10.3390/molecules24091734 (PMC6540195; doi:10.3390/molecules24091734)
Supplement: Supplementary file 1 [file molecules-24-01734-s001.pdf]

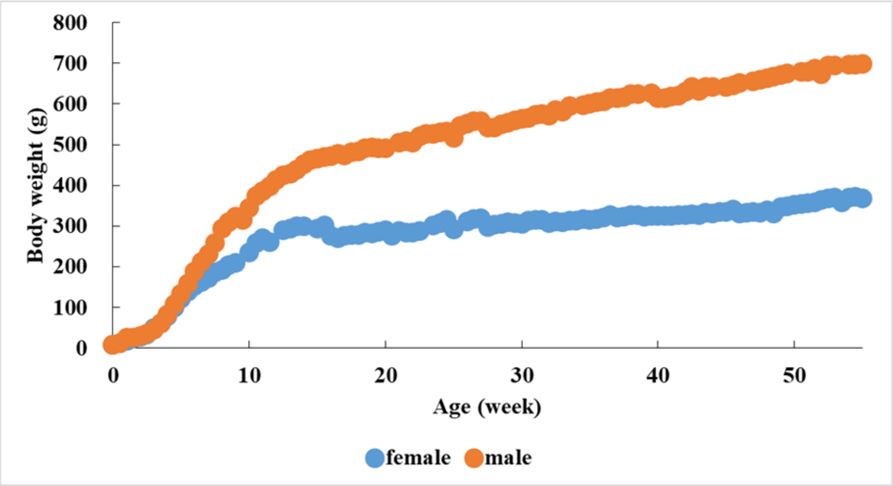

Figure S1: The growth curve of P1-HT rats.

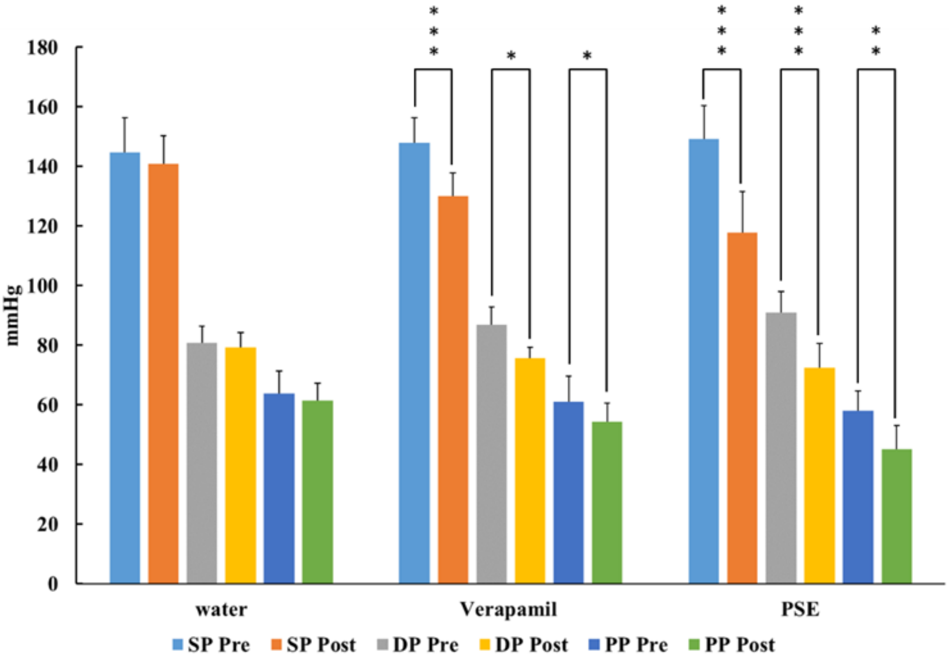

Fig. S2: The rats were orally gavaged with water as a control treatment, the blood pressure was no significant different between Pre and Post-treatment. The rats were orally gavaged with verapamil could significant decrease on the systolic pressure (SP), diastolic pressure (DP), and pulse pressure (PP). The rats orally gavaged with PSE caused significant decrease on the systolic pressure (SP), diastolic pressure (DP), and pulse pressure (PP). (* $p<0.05$, ** $p<0.01$, *** $p<0.001$)

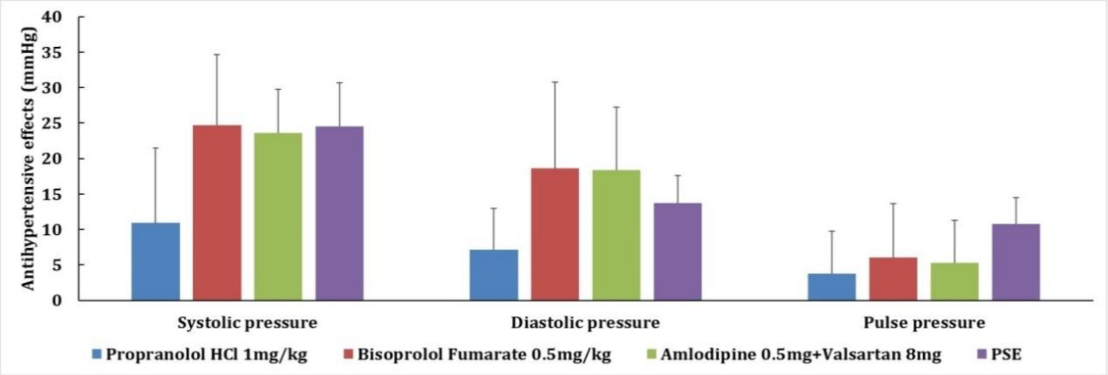

Fig. S3. The antihypertensive effects of different types of clinical drugs and *Plantago asiatica* seed Extracts (PSE) on P1-HT rats (n=5~6). The hypotensive effects of PSE are comparable to current clinical antihypertensive medications.
